# Supplementary material for: A New Cryptic Lineage in Parmeliaceae (Ascomycota) with Pharmacological Properties
Source: J Fungi (Basel). 2022 Aug 8;8(8):826. doi: 10.3390/jof8080826 (PMC9409757; doi:10.3390/jof8080826)
Supplement: Supplementary file 1 [file jof-08-00826-s001.zip › Figure S1 Relative amounts of the main metabolites in 13 Canoparmelia samples.pdf]

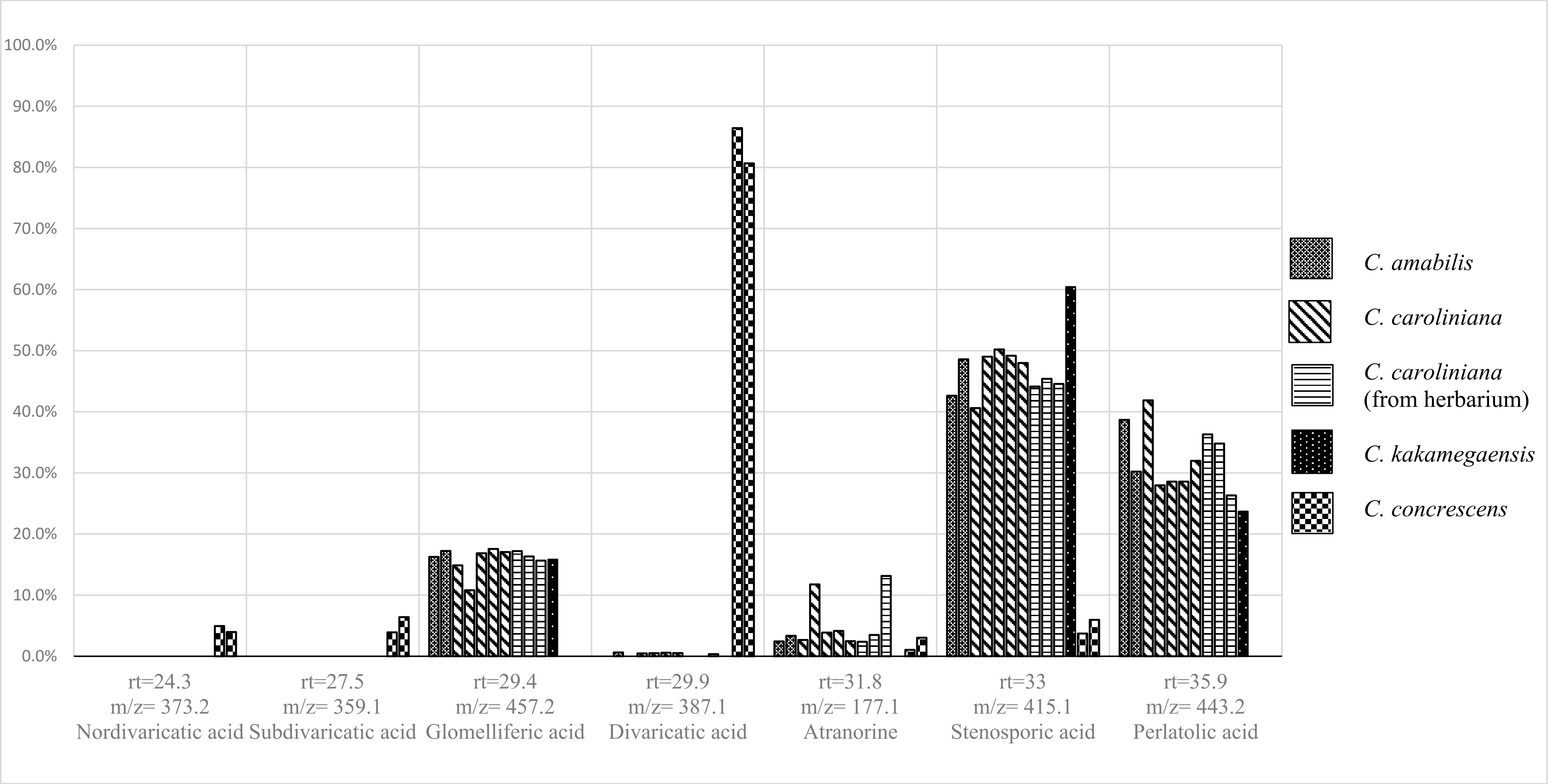

Figure S1. HPLC features (rt, m/z) of the major detected metabolites with their relative amounts in the 13 sample thalli analyzed (% calculated from 254 nm absorbances)
