# Supplementary material for: A New Cryptic Lineage in Parmeliaceae (Ascomycota) with Pharmacological Properties
Source: J Fungi (Basel). 2022 Aug 8;8(8):826. doi: 10.3390/jof8080826 (PMC9409757; doi:10.3390/jof8080826)
Supplement: Supplementary file 1 [file jof-08-00826-s001.zip › Table S1 C caroliniana_Revised_01.08.22.pdf]

Table S1. Specimens of *Canoparmelia* used in this study, including sample code, collection details, voucher and GenBank Accession numbers. Newly obtained sequences for this study are in bold and missing data are indicated with a dash (—).

**Taxon label**	**Locality**	**Collector(s)**	**Voucher specimen**	**GenBank Accession numbers**		
				nuITS	mtSSU	nuLSU
2301_*Canoparmelia austroamericana*	Argentina	Michlig & Niveiro	Michlig 2301	KY929408	-	-
6515_ *Canoparmelia* cf. *concrescens*	Kenya: West Pokot County, Kapenguria	Kirika, 5233A	EA	**OP046386**	**-**	**OP046382**
4688_*Canoparmelia amabilis*	Portugal: Azores Island, San Jorge, Fgada das Cubres,	Divakar 4688	MAF-Lich 24553	**OP046387**	**OP046401**	**OP046383**
4682_*Canoparmelia amabilis*	Portugal: Azores Island, San Jorge, Parque Sete Fontes,	Divakar 4682	MAF-Lich 24554	**OP046388**	-	**-**
1_*Canoparmelia caroliniana*	USA: North Carolina	Perlmutter 1000	NCU	GU994542	AY584613	GU994584
25_*Canoparmelia caroliniana*	USA	AFTOL-ID 6	NCU	-	-	AY584634
4680_*Canoparmelia caroliniana*	Portugal: Azores Island, San Jorge, Fgada das Cubres,	Divakar 4680	MAF-Lich 24555	**OP046389**	-	**OP046384**
2_*Canoparmelia caroliniana*	Kenya: Kakamega County, Kakamega forest, Isecheno Forest Station	Kirika, 3419	EA, F	KX369243	-	KX369261
3_*Canoparmelia caroliniana*	Kenya: Kakamega County, Kakamega forest, Buyangu,	Kirika, 3389	EA, F	KX369244	KX369256	KX369262

	Edeos Campsite					
9_*Canoparmelia concrescens*	Kenya: Kakamega County	Divakar, Mangold & Lumbsch 19538f	MAF-Lich 15547	GU994543	KR995317	GU994585
6545_*Canoparmelia texana*	Kenya: Kakamega County, Lirondo Hill	Kirika, 5232	EA, MAF	OK561343	OK582193	OK561867
5_*Canoparmelia ecaperata*	Kenya: Makueni County	Kirika, Malombe & Matheka, 3692	EA, F	KX369246	-	KX369264
6_*Canoparmelia eruptens*	Kenya: Taita Taveta County, Ngangao Forest	Kirika, Mugambi & Lumbsch, 2405	EA, F	KX369247	-	
7_*Canoparmelia eruptens*	Kenya: Taita Taveta County, Ngangao Hill	Kirika, 4483	EA, F, MAF	KX369248	KX369257	KX369265
12_*Canoparmelia nairobiensis*	Kenya: Kitui County, Mwingi, Nuu Hill	Kirika & Lumbsch, 3866	EA, MAF, F	GU994545	KR995318	KX369268
13_*Canoparmelia nairobiensis*	Kenya: Nyeri County, Mt. Kenya, Gathiuru Forest, Naromoru	Kirika, 4423	EA, MAF, F	KX369252	KX369259	KX369269
16_*Canoparmelia texana*	India: Uttaranchal	Divakar GPGC 02-000637	MAF-Lich 14272	EF042906	-	EF042915
6518_*Canoparmelia texana*	Kenya: Kakamega County, Lirondo Hill	Kirika, 5232A	EA, MAF	**OP046390**	**OP046402**	**OP046385**
18_*Xanthoparmelia chlorochroa*	USA: North Dakota	Leavitt 55437	BRY-C	HM578887	KR995372	HM579298

19_*Xanthoparmelia conspersa*	Spain: Zamora	Blanco & Crespo s.n.	MAF-Lich 6793	AY581096	AF351186	AY578962
