# Supplementary material for: A New Cryptic Lineage in Parmeliaceae (Ascomycota) with Pharmacological Properties
Source: J Fungi (Basel). 2022 Aug 8;8(8):826. doi: 10.3390/jof8080826 (PMC9409757; doi:10.3390/jof8080826)
Supplement: Supplementary file 1 [file jof-08-00826-s001.zip › Table S2. List of Canoparmelia taxa for chemical profiling.pdf]

TableS2. List of *Canoparmelia* taxa used for chemical profiling.

1. *Canoparmelia amabilis*: USA, North Carolina, MAF-Lich 19833, Collection date, 2010
2. *Canoparmelia amabilis*: Portugal, Azores Is., Coll. No. 6907K, MAF-Lich 24554, Collection date, 2012
3. *Canoparmelia caroliniana*: USA, North Carolina, MAF-Lich 19832, Collection date, 2010
4. *Canoparmelia caroliniana:* Ecuador, Galapagos, CDRS herb no. 28937, Bungartz coll. no. 4805, Collection date, 2006
5. *Canoparmelia caroliniana:* Portugal: Azores Is., Coll. No. 6904A, MAF-Lich, Collection date, 2011
6. *Canoparmelia caroliniana:* Portugal: Azores Is., Coll. No. 6909E, MAF-Lich, Collection date, 2011
7. *Canoparmelia caroliniana:* Portugal: Azores Is., Coll. No. 6909D, MAF-Lich 24555, Collection date, 2011
8. *Canoparmelia caroliniana:* USA,Virginina, REN-Abb, M. E. Hale, n°12771, Collection date, 1957
9. *Canoparmelia caroliniana:* Mexico, Chiapas, REN-Abb, M. E. Hale, n°20104, Collection date, 1960
10. *Canoparmelia caroliniana:* Dominican Republic, REN-Abb, H.A. Imshaugh, n°2378, Collection date, 1958
11. *Canoparmelia concrescens:* Kenya, P. Kirika coll. No. 4477, EA, Collection date, 2014
12. *Canoparmelia concrescens:* Kenya, P. Kirika coll. No. 3692, EA, Collection date, 2013
13. *Canoparmelia kakameagensis:* Kenya, P. Kirika coll. No. 3419, EA, Collection date, 2013
