# Supplementary material for: A New Cryptic Lineage in Parmeliaceae (Ascomycota) with Pharmacological Properties
Source: J Fungi (Basel). 2022 Aug 8;8(8):826. doi: 10.3390/jof8080826 (PMC9409757; doi:10.3390/jof8080826)
Supplement: Supplementary file 1 [file jof-08-00826-s001.zip › Table S3. IC50 (μg_mL) values of MCF-7 and HepG2 cells after 24 h incubation.pdf]

Table S3. IC_{50} (µg/mL) values of MCF-7 and HepG2 cells after 24 h incubation with the methanol extracts of *Canoparmelia kakamegaensis* and *Canoparmelia caroliniana*. Values are expressed as mean ± standard deviation. *Indicates statistically significant differences ($p < 0.05$) between lichen extracts.

	IC_{50} values (µg/mL)	
**Extracts**	**MCF-7 cells**	**HepG2 cells**
Canoparmelia kakamegaensis	*38.2 ± 3.4	*34.3 ± 5.1
Canoparmelia caroliniana	43.3 ± 3.5	45.3 ± 3.0
